# Evaluation of Compliance With International Guidelines During the Follow-Up of Hypoparathyroidism

**DOI:** 10.1155/ije/3174295

**Published:** 2025-05-05

**Authors:** Beril Turan Erdogan, Sefika Burcak Polat, Caglar Keskin, Narin Nasiroglu İmga, Didem Ozdemir, Oya Topaloglu, Reyhan Ersoy, Bekir Cakir

**Affiliations:** ^1^Department of Endocrinology and Metabolism, Ankara Bilkent City Hospital, University of Health Sciences, Ankara, Türkiye; ^2^Department of Endocrinology and Metabolism, Ankara Yildirim Beyazit University School of Medicine, Ankara, Türkiye

**Keywords:** complications, ESE hypoparathyroidism guideline, guidelines, hypoparathyroidism

## Abstract

**Aims:** Hypoparathyroidism is a disorder that causes renal complications, bone disease, an increased risk of cardiovascular diseases, neuromuscular complications, and ocular findings. In this study, we aimed to determine how well the existing guidelines were followed in the follow-up of patients with hypoparathyroidism.

**Materials and Methods:** In the endocrinology and metabolism outpatient clinic, routine controls were conducted on patients 18 years of age and older with a diagnosis of hypoparathyroidism between February 2019 and September 2021. These individuals' test findings, imaging, hormones, and additional disorders were assessed.

**Results:** The mean age of 264 patients included in the study was 49.34 ± 12.98 (22–91). Of the patients, 211 (79.9%) were female. Blood biochemistry tests were performed on all patients. Ophthalmological examination was performed in 3% of the patients, brain imaging in 9.8%, renal imaging in 18.6%, and 24-h urinary Ca measurements in 11%. According to the results of the examinations, cataract was found in 2 (0.8%) patients and basal ganglia calcification was found in 4 (1.5%) patients. In addition, nephrocalcinosis or nephrolithiasis was seen in 2 patients (0.8%). Hypercalciuria was defined as a 24-h urinary calcium excretion of > 300 mg/24 h for males and > 250 mg/dL for females and was detected in 12 patients with hypoparathyroidism.

**Conclusion:** In our research, it was discovered that patients with hypoparathyroidism had low compliance rates with the guidelines. We believe that the cause of this is the inability to provide the patients with enough time in intense polyclinic conditions. Given the information gathered from our study, it has been concluded that complication screening in hypoparathyroidism patients is just as crucial as blood tests and that allocating enough time for patients will enhance their follow-up and treatment.

## 1. Introduction

Hypoparathyroidism (HypoPT) is an uncommon condition characterized by undetectable or inappropriately normal levels of PTH measured by a 2nd or 3rd generation test twice at least 2 weeks apart leading to hypocalcemia and hyperphosphatemia [1, 2]. The primary cause of hypoPT is the accidental removal of the parathyroid glands during thyroid or parathyroidectomy surgeries [3, 4]. Chronic hypoPT presents with various clinical manifestations, including renal complications such as nephrolithiasis and impaired kidney function, reduced bone turnover and quality, an increased risk of cardiovascular disease and cataracts, impairment of the neuromuscular system, and neuropsychological symptoms that negatively impact the quality of life [5]. Numerous investigations, conducted in diverse geographical locations, have explored the prevalence of persistent hypoPT. A comprehensive review of published studies reveals a range in the reported prevalence of chronic hypoPT, spanning from 6.4 to 37 per 100,000 population across various countries. These studies, originating from different regions, contribute valuable insights into the epidemiology of persistent hypoPT, highlighting the variability in its occurrence on a global scale [6–8].

Due to its potential life-threatening consequences and negative impact on quality of life, hypoPT requires careful and systematic follow-up. The European Society of Endocrinology (ESE) published the clinical guideline: Treatment of Chronic Hypoparathyroidism in Adults in 2015, which serves as the most commonly utilized guideline for monitoring hypoPT in Europe and Turkey [9]. Additionally, the management of hypoPT is guided by the summary statement and guidelines released in 2016 [1], as well as the Canadian and international consensus on standards of care for hypoPT in adults, which is an updated version of the previous guide [10].

According to hypoPT guidelines, it is recommended to annually or more frequently check the levels of calcium, phosphorus, albumin, magnesium, creatinine, and estimated glomerular filtration rate (eGFR) as part of patient follow-up. Additionally, a 24-h urine calcium (uCa) excretion test should be conducted once a year. Renal imaging is advised for patients with elevated creatinine levels, high urinary calcium excretion, or symptoms suggestive of renal stones. For basal ganglia calcification, a brain MRI or CT scan is recommended. Furthermore, an annual eye examination is advised to check for the presence of cataracts [1, 9, 10].

Our research aimed to investigate whether the guidelines for long-term follow-up of patients with hypoPT were being adhered to. By conducting this study, we aimed to enhance the understanding of the appropriate practices for monitoring patients with hypoPT.

## 2. Materials and Methods

Our study was designed as an observational, single-center study with a retrospective approach. It included patients with hypoPT who had undergone routine scheduled monitoring visits at the Endocrinology Department of Ankara City Hospital between February 2019 and September 2021. The study enrolled adult individuals of both genders, aged 18 years or older, who had chronic hypoPT. Chronic hypoPT was defined as low serum calcium levels accompanied by persistently low or inappropriately normal parathyroid hormone (PTH) levels for a minimum of 12 months prior to enrollment. While earlier research indicated a 6-month duration for permanent hypoPT, recent studies have established this period as 12 months. In our study, we adhered to the updated findings, considering 12 months as the recognized timeframe for permanent hypoPT [11]. Inclusion criteria also required that patients had been receiving conventional therapy, including calcium supplements and active vitamin D metabolites, for at least 12 months before their inclusion in the study.

We collected data on patients' demographics, including age, age of onset, gender, and duration of the disease. Information regarding the etiology of hypoPT and current pharmacologic therapies was also obtained. The study included analysis of the patients' biochemical profile, including serum calcium (Ca), albumin, serum phosphorus (P), intact parathyroid hormone (iPTH), alkaline phosphatase (ALP), 25-hydroxy vitamin D, creatinine (Cr), magnesium (Mg), and 24-h uCa excretion. Additionally, we recorded data on renal imaging, brain computed tomography (CT) scans or magnetic resonance imaging (MRI), eye examinations, and bone mineral density (BMD) results. Information regarding hospitalization related to hypoPT was also collected.

Serum calcium (Ca), albumin, phosphorus (P), magnesium (Mg), ALP, creatinine (Cr), and 24-h uCa levels were quantified using standard laboratory techniques. To account for variations in albumin levels, adjusted calcium (Adj Ca) levels were calculated using the formula: Adj Ca = Serum Ca + (0.8 × [normal albumin − patient albumin]). Plasma iPTH levels were measured using the Allegro IRMA assay from Roche Diagnostics. The normal range for iPTH was 15–60 pg/mL, with a detection limit of 1 pg/mL. The intra-assay and interassay coefficients of variation were 2% and 10%, respectively. The levels of 25-hydroxyvitamin D were determined using liquid chromatography and tandem mass spectrometry (Shimadzu-API LC-MS-MS API 3200, Canada), with a normal range of 20–80 ng/L.

Renal ultrasonography (US) or abdominal CT scans were used to assess the presence of nephrolithiasis in the patients. Evaluation of basal ganglia calcification in the brain was performed using either CT scans or MRI. Ophthalmologists conducted eye examinations, classifying patients as “normal,” “cataract,” or “glaucoma.” BMD was measured using dual-energy X-ray absorptiometry (DEXA) with a QDR-4500 machine from Hologic Inc, Waltham, MA. DEXA measurements were taken for the lumbar vertebrae and femur. BMD results were expressed as *T* scores or *Z* scores. The diagnosis of osteoporosis was determined based on the region with the lowest *T* score in the lumbar spine and femoral neck, in accordance with the criteria set by the World Health Organization (WHO). The results were classified into categories including osteoporosis, osteopenia, low bone mass (LBM) for chronological age, and normal.

### 2.1. Statistical Analysis

SPSS 25.0 Statistical Package Program for Windows (IBM Corp., Armonk, NY, USA) was used for statistical analyses. Descriptive summaries are presented as mean  ± standard deviation (SD) or as median and intervals for continuous variables and counts and percentage (%) for categorical data. For subgroup comparisons between postsurgical and nonsurgical hypoPT, independent samples *t* test or Mann–Whitney *U* test was used for continuous variables depending on the distribution, and the chi-square or Fisher's exact test was used for categorical variables.

## 3. Results

From February 2019 to September 2021, a total of 264 patients with persistent hypoPT were included in the study. The average age of the patients was 49.34 years, ranging from 22 to 91 years. The mean age of onset of hypoPT was 40.34 years. The average duration of hypoPT among the participants was 7.5 years. Of the enrolled patients, 79.9% were female (*n* = 211). The mean interval between visits was 5.11 months, ranging from 1 to 12 months, and the average number of requested parameters per visit was 6.67, ranging from 4 to 9. Medications remained unchanged in 51.1% of patients (*n* = 135). Among the participants, 86% (*n* = 227) had postsurgical hypoPT, while 14% (*n* = 37) had nonsurgical hypoPT, with idiopathic hypoPT being the most common cause (Table 1).

A subgroup analysis comparing postsurgical (*n* = 227) and nonsurgical (*n* = 37) hypoPT patients revealed several notable differences. In the postsurgical group, the mean corrected serum calcium level (8.66 ± 0.77 mg/dL) was significantly higher than that in the nonsurgical cohort (8.17 ± 0.99 mg/dL; *p*=0.006). Likewise, mean PTH levels were significantly higher in postsurgical patients (16.92 ± 13.37 pg/mL) compared with nonsurgical cases (9.57 ± 8.23 pg/mL; *p* < 0.001). By contrast, serum phosphate levels did not differ statistically between the two groups (4.57 ± 0.68 mg/dL vs. 4.88 ± 0.61 mg/dL; *p*=0.060). Glomerular filtration rates (GFRs) were marginally higher among nonsurgical patients (100.36 ± 26.00 vs. 90.98 ± 20.30 mL/min/1.73 m^2^; *p*=0.045). In the small subset of individuals (*n* = 29) with 24-h urinary calcium measurements, no significant group difference was observed.

Because imaging studies (brain MRI/CT, renal ultrasound/CT, and ophthalmologic examinations) were not routinely performed in all patients, the available data on complications were limited. Nevertheless, among the small number of patients with brain imaging, basal ganglia calcification was more prevalent in those with nonsurgical hypoPT (3/9; 33.3%) than in postsurgical patients (1/17; 5.9%). In contrast, nephrolithiasis or nephrocalcinosis was observed only in the postsurgical subgroup (2/38; 5.3%), whereas none of the nonsurgical patients who underwent renal imaging (0/11) had these abnormalities. Similarly, among four ophthalmologically evaluated patients in each subgroup, one nonsurgical case (25%) and two postsurgical cases (50%) exhibited cataract or other ocular abnormalities. BMD measurements, performed in only a few patients, indicated osteopenia or osteoporosis in the majority of both groups (> 90%); however, this rate may not reflect the true frequency due to the limited number of assessments. Although these findings suggest potential etiologic differences between postsurgical and nonsurgical hypoPT, the lack of universal imaging/testing and the imbalance in subgroup sizes constrain any definitive conclusions.

Among patients with postsurgical hypoPT, 97.8% (222 out of 227) had undergone thyroidectomy, while only 2.2% (5 out of 227) had minimally invasive parathyroidectomy. Out of the total operated patient population, 53.2% (118 patients) had benign thyroid pathology results, while 46.8% (104 patients) had malignant pathology results. Specifically, papillary thyroid carcinoma was the most common malignant pathology, observed in 95 individuals. When the follow-up intervals were compared between patients with malignant and benign thyroid pathology, there was no statistically significant difference (5.26 ± 2.30 vs. 4.84 ± 1.48 months, respectively; *p*=0.510), and similarly, no remarkable difference was observed in the frequency of complication screenings between the two groups.

Among patients with chronic hypoPT, the mean albumin-corrected serum calcium level was 8.19 ± 0.60 mg/dL (range 6.25–9.88 mg/dL). Approximately 32.6% of patients had serum calcium levels within the normal reference range, while 67.4% of patients had serum calcium levels below 8.5 mg/dL. The mean serum phosphate level was slightly elevated, measuring 4.56 ± 0.75 mg/dL (range 2.50–7.20 mg/dL). Among the patients, 17.8% had elevated serum phosphate levels, exceeding 5.1 mg/dL.

Among patients with hypoPT, approximately 72.1% had serum 25-hydroxy vitamin D levels below 30 ng/dL. The mean serum 25-hydroxy vitamin D level was 25.41 ± 8.86 ng/dL, ranging from 5.00 to 57.00 ng/dL. Hypercalciuria, defined as a 24-h uCa excretion exceeding 300 mg/24 h, was observed in 27.6% of the participants with hypoPT. The mean 24-h uCa excretion was 203.01 ± 117.98 for females and 214.16 ± 135.91 mg/24 h for males. Further details regarding other laboratory values can be found in Table 2.

Among the 264 patients included in the study, 255 (96.6%) were not using medications known to induce hypocalcemia, while 9 (3.4%) were on at least one such as steroids, bisphosphonates, and diuretics. The most commonly reported comorbidities were hypertension, Type 2 diabetes mellitus, cardiovascular diseases, and rheumatologic diseases. The most frequently used medications included antihypertensive medications, oral antidiabetics, and statins.

All 264 patients in the study were receiving calcium supplements as part of their treatment. Among them, 9 patients were taking a combination of calcium carbonate and calcium acetate. The majority of patients, 233 (88.3%), were also receiving calcitriol. A small percentage, approximately 7.2% (*n* = 19), were prescribed hydrochlorothiazide. It is worth noting that none of the patients with uCa levels exceeding 250 mg/h for females or 300 mg/24 h for males were on hydrochlorothiazide. Additionally, 3 patients were using sevelamer, and another 3 patients were using PTH as part of their treatment.

Out of the 264 patients, only 3% (*n* = 8) completed eye examinations, with seven of them being requested by the endocrine department. Among the patients who underwent eye examinations, cataracts were observed in two individuals (0.8%) with postsurgical hypoPT.

A total of 26 patients (9.8%) underwent brain imaging using either CT or MRI scans, and nine of these exams were requested by the endocrine department. Basal ganglia calcification was detected in 4 patients (1.5%). Among these patients, 1 had postsurgical hypoPT, while the remaining 3 had nonsurgical hypoPT.

Forty-nine patients underwent renal imaging, with eight of them having the exams requested by the endocrine department. Nephrocalcinosis or nephrolithiasis was observed in 2 patients (0.8%), both of whom had postsurgical hypoPT. Unfortunately, these patients did not have 24-h urinary calcium results available. A total of 29 patients (11%) had 24-h urinary calcium results, with 23 of them having the exams requested by the endocrine department. BMD measurements were performed on 22 patients (8.3%), with 10 of them having the exams requested by the endocrine department. None of these patients had a history of fractures. One patient was diagnosed with osteoporosis, while seven others had osteopenia. These individuals all had postsurgical hypoPT. One patient with nonsurgical hypoPT was found to have LBM for chronological age (Table 3).

Calcium, phosphorus, and magnesium levels were assessed in the entire cohort of 264 participants enrolled in the study, with the corresponding distribution of patients meeting the target values outlined in Table 4. In contrast, the measurement of 24-h urinary calcium excretion was limited to only 6 male and 23 female patients. Consequently, the analysis revealed a noteworthy significance in achieving four out of 5 target values as per the existing literature. Furthermore, the examination of complication rates in patients was contingent on the attainment of predefined targets; however, due to the relatively low number of patients undergoing assessment for complications, statistical significance could not be established.

Out of the 264 patients, 13 (4.9%) required hospitalization during the study period. The most common reason for hospitalization was symptomatic hypocalcemia, accounting for 61.5% (*n* = 8) of the cases. Additionally, one patient was hospitalized due to hypercalcemia, while two patients were admitted for tetany and two others for hyperglycemia.

## 4. Discussion

HypoPT is a disorder characterized by low levels of calcium and high levels of phosphorus, leading to various complications affecting the kidneys, bones, cardiovascular system, neuromuscular system, and eyes. Given the wide-ranging effects of the condition, it is essential to monitor patients carefully and systematically. Clinicians currently rely on established guidelines to guide their approach. These guidelines, including the ESE guideline, provide standardized criteria for the follow-up of hypoPT globally.

In our study, we aimed to evaluate the extent to which these existing guidelines were followed in the follow-up of patients with hypoPT. Our findings revealed that many of the recommended screenings and examinations for potential complications were not adequately conducted, despite being mentioned in the guidelines. Specifically, only a small percentage of patients, 3%, underwent the recommended eye examination. Similarly, brain imaging was performed in only 9.8% of patients, renal imaging in 18.6%, and BMD assessment in 8.3%. These results highlight the need for improved adherence to the recommended guidelines in the management and follow-up of patients with hypoPT. Ensuring comprehensive monitoring and screening can help detect and manage potential complications in a timely manner, ultimately improving patient outcomes.

Upon examining the secondary findings of our study, we discovered significant gaps in the detection of complications among patients. Among the patients who underwent eye scans, 37.5% (3 out of 8) were found to have cataracts or glaucoma. Similarly, calcification was observed in the basal ganglia of 15.4% (4 out of 26) of patients who underwent brain scans. Renal stones were detected in 4.1% (2 out of 49) patients who underwent renal imaging, and osteopenia or osteoporosis was found in 36.4% (8 out of 22) patients with BMD assessments.

When extrapolating these findings to the entire study population, it is estimated that if all patients had undergone the recommended scans, ocular findings would have been identified in 99 patients, renal stones in 10 patients, basal ganglia calcification in 40 patients, and osteopenia or osteoporosis in 97 patients. This suggests that among the 264 individuals included in the study, a significant number of cases (91 eye diseases, 36 brain calcifications, 8 kidney stones, and 89 cases of osteopenia or osteoporosis) went undiagnosed.

Our study identified significant gaps in the detection of complications among patients with hypoPT. For example, only a small percentage of patients underwent recommended scans, resulting in undiagnosed ocular diseases, brain calcifications, renal stones, and osteopenia/osteoporosis. Early intervention based on these findings could potentially improve patients' quality of life.

Another thing to consider is that all our patients were receiving standard therapy for hypoPT, including calcium supplements (and in most cases active vitamin D). While this approach controls hypocalcemia and improves clinical outcomes, it may also mask or modify certain laboratory findings and complication rates.

Interestingly, our cohort demonstrated a relatively high rate of achieving the recommended target values for biochemical parameters such as serum calcium, phosphorus, magnesium, and calcium–phosphate product. This finding may be related to the shorter mean follow-up intervals (5.1 months), consistent treatment protocols, and regular monitoring in a specialized tertiary center. Moreover, the majority of patients were receiving both calcium and active vitamin D supplements, which likely contributed to maintaining optimal biochemical control. However, this result may not reflect general practice, and caution is needed in generalizing these findings to other healthcare settings.

As a result, direct correlations between hypoPT itself and various outcome measures can be less apparent because the treatment—rather than the underlying disease—becomes a dominant influence on serum calcium, phosphate, and other parameters. Nevertheless, this reflects real-life clinical practice, where virtually all hypoparathyroid patients receive supplementation. Future studies that include comparison with treatment-naïve or minimally treated patients, or that focus on different treatment regimens (e.g., PTH analogs), may provide additional insights into the natural course of hypoPT and its direct correlations with clinical and biochemical findings.

Although postsurgical and nonsurgical hypoPT share the hallmark features of hypocalcemia and hyperphosphatemia, subtle differences exist between these subgroups. Our findings align with previous studies showing that postsurgical patients tend to have slightly higher mean serum calcium and PTH levels compared with nonsurgical cases, likely due to partial preservation of parathyroid function in some individuals [11]. In contrast, nonsurgical patients often exhibit more profound PTH deficiency, especially in autoimmune and genetic forms, which may contribute to greater difficulty in maintaining stable calcium levels [12]. Renal complications are a significant concern in both groups, driven by chronic hypercalciuria and calcium–phosphate imbalance. While postsurgical patients show a higher prevalence of nephrolithiasis, likely related to increased calcium supplementation, nonsurgical patients may be at greater risk for progressive CKD, possibly due to lifelong hyperphosphatemia and calcium deposition in renal tissue [13]. Similarly, basal ganglia calcifications appear to be more frequent in nonsurgical patients.

In a systematic literature review conducted by Gosmanova et al. in 2021, nephrocalcinosis was reported in up to 38% of patients with hypoPT under conventional treatment. The prevalence of chronic kidney disease attributed to renal stones ranged from 2.5% to 41% [14]. Meola et al. [15] found kidney stones in 30% of 90 patients with primary hyperparathyroidism, while Hadker et al. [16] reported a rate of 35.5% in a study of 374 patients. Mitchell et al. [17] observed renal calcification in 31% of patients who underwent renal imaging and basal ganglia calcification in 52% of patients. The ESE guidelines recommend renal imaging for patients with symptoms of renal stones or elevated creatinine, as well as monitoring of 24-h urinary calcium excretion [9].

The Canadian and International Consensus Guidelines recommend screenings and imaging for detecting renal complications and basal ganglia calcification in patients with hypoPT. However, our study found low adherence to these guidelines, with only a small number of patients undergoing renal imaging and minimal cases of renal calcification detected. This highlights the need for improved compliance with recommended imaging protocols. Insufficient monitoring of 24-h urinary calcium may have contributed to delays in identifying patients who require imaging. Adhering to the guidelines by implementing regular urinary calcium monitoring and annual renal imaging can facilitate early detection of complications such as renal stones and basal ganglia calcification [1]. Furthermore, renal imaging can assist in diagnosing and managing related conditions, such as HDR syndrome, an autosomal dominant syndrome [18].

The connection between hypoPT and cataract formation was first proposed by Haft in 1953 [19]. Subsequent studies by Stein, Eytan, and Akaichi further supported this association, reporting cases of cataract development in patients with hypoPT. The relationship between glaucoma and hypoPT remains unclear, although a patient with autoimmune polyglandular syndrome Type 1 was found to have both bilateral cataracts and glaucoma [20–22]. The ESE guidance advises regular monitoring for cataract development in patients with chronic hypoPT, and the Canadian and International Consensus Guidelines recommend annual eye exams for cataracts. However, in our study, only eight out of 264 patients underwent ophthalmologic examination, revealing 2 cases of cataract and 1 case of glaucoma. We believe that if the guidelines were followed more closely, a higher number of eye problems could have been detected and addressed earlier.

The ESE guidelines do not recommend routine monitoring of BMD [9]. Similarly, the Canadian and International Consensus Guidelines do not recommend BMD screening, although they acknowledge that osteomalacia and decreased quality of life due to osteoporosis may occur [1]. However, the “Management of Hypoparathyroidism: Summary Statement and Guidelines” suggests BMD screening when clinically indicated [10]. In our study, a significant proportion (36.4%) of patients had osteopenia or osteoporosis, and only 8.3% of patients underwent BMD testing. This high rate of bone disease suggests that it would be appropriate to perform a BMD scan at least once during the diagnosis phase and consider repeat examinations when clinically necessary to ensure early detection and management of bone-related complications in patients with chronic hypoPT.

The management of permanent hypoPT underscores the pivotal significance of achieving specific laboratory targets and the prevention of complications. In the course of our investigation, a comprehensive examination of the majority of patients' laboratory parameters was conducted; however, scrutiny of 24-h urinary calcium excretion was confined to a subset of patients with limited representation. Furthermore, the assessment of complications was restricted to a notably small cohort. As highlighted previously, nonadherence to established guidelines not only poses challenges in patient monitoring and therapeutic interventions but also results in a dearth of scientific prognostication for future considerations. While physician workload and time constraints are certainly major contributors to suboptimal adherence to hypoPT follow-up guidelines, other factors may also play a role. These include patient-related barriers such as noncompliance with referrals, limited understanding of the importance of follow-up examinations, and socioeconomic challenges that may limit access to specialized imaging or consultations. Additionally, health system limitations such as appointment scheduling issues, referral bottlenecks, or insufficient coordination between departments could further impact adherence to recommended monitoring protocols. It is crucial to recognize that adherence to guidelines plays a dual role: not only optimizing patient care and follow-up but also contributing significantly to the generation of scientific knowledge, thereby advancing the understanding and treatment of permanent hypoPT. Consequently, the integration of guidelines into clinical practice not only ensures the effective management of patients but also fosters the growth of evidence-based insights for the broader medical community.

Our study has several limitations that need to be acknowledged. First, its retrospective design introduces inherent biases and limitations associated with data collection and analysis. Additionally, being conducted at a single center may limit the generalizability of our findings to a broader population.

Another potential limitation of our study is the bias introduced by comorbidities and concomitant medications that may alter serum calcium and related laboratory findings [4, 23]. In our cohort, only nine of the 264 patients (3.4%) were receiving agents known to induce hypocalcemia (e.g., steroids or loop diuretics), and we systematically noted medication changes at each visit. Although several participants had comorbidities such as hypertension or diabetes mellitus that can also affect biochemical parameters, the proportion with advanced disease states was relatively small. Therefore, while these factors reflect real-world clinical practice and cannot be fully eliminated, we believe their impact on our overall results was minimal.

However, there are also notable strengths to consider. One of the strengths is the large number of patients included in the study, which is significant considering the rarity of the disease. This allows for a more comprehensive evaluation of the population under study. Furthermore, to the best of our knowledge, our study is unique in its focus on assessing compliance with the guidelines, making it a valuable contribution to the existing literature on this topic.

## 5. Conclusion

In summary, hypoPT necessitates regular and comprehensive follow-up to manage its chronic complications. However, our study revealed a low level of compliance, with less than 10% adherence to international guideline recommendations among clinicians. This lack of compliance may be attributed to time constraints in outpatient clinic settings, where adequate time allocation for each patient is challenging. Other contributing factors, such as limited patient adherence or socioeconomic barriers, may also hinder proper follow-up. To improve patient follow-up, it is crucial for health authorities to create conditions that allow for sufficient time to be dedicated to each patient.

## Figures and Tables

**Table 1:** Demographics, etiology of hypoparathyroidism.

	Total (*n* = 264)
Age, years	49.34 ± 12.98
Age at onset of hypoparathyroidism, years	40.34 ± 13.21
Duration of hypoparathyroidism, years	7.50 ± 6.68
Gender	
Female (%)	211 (79.9)
Male (%)	53 (20.1)
Visits interval, months	5.11 ± 2.01
Parameter	6.67 ± 0.80
Medication change, *n* (%)	
Never	135 (51.1)
Once	108 (40.9)
Twice	17 (6.4)
Three times	3 (1.1)
Four times	1 (0.4)
Etiology	
Postsurgical hypoparathyroidism	227 (86)
Nonsurgical hypoparathyroidism	37 (14)
Idiopathic	31 (11.7)
Hemochromatosis	3 (1.1)
Autoimmune	2 (0.8)
Genetic variants-CASR gene mutation	1 (0.4)

*Note:* Data are presented as mean ± SD or number (%).

**Table 2:** Biochemical profile of patients.

	Result	Reference range
Creatinine, mg/dL	0.84 ± 0.50	0.7–1.3
GFR, mL/min/1.73 m^2^	91.52 ± 20.11	
Calcium, mg/dL	8.62 ± 0.61	8.7–10.4
Albumin, g/L	45.33 ± 2.50	32–48
Phosphorus, mg/dL	4.56 ± 0.75	2.5–5.1
Magnesium, mg/dL	1.80 ± 0.23	1.3–2.7
Alkaline phosphatase, U/L	71.04 ± 27.17	56–119
Parathormone, ng/L	15.4 ± 11.74	15–60
25-OH D vit, ng/mL	25.41 ± 8.86	30–150
Corrected calcium, mg/dL	8.19 ± 0.60	8.5–10.5
24-h urinary Ca, mg/24 h for male	214.16 ± 135.91	100–300
24-h urine Ca normal (< 300 mg/24 h)	4/6 (66.7)	
24-h urine Ca elevated (> 300 mg/24 h)	2/6 (33.3)	
24-h urinary Ca, mg/24 h for female	203.01 ± 117.98	100–250
24-h urine Ca normal (< 250 mg/24 h)	13/23 (56.5)	
24-h urine Ca elevated (> 250 mg/24 h)	10/23 (43.5)	

*Note:* Data are presented as mean ± SD or as number (percentage).

**Table 3:** Hypoparathyroidism complication screening.

	Total (*n* = 264)
Eye screening	8 (3)
Normal	5 (1.9)
Cataract	2 (0.8)
Glaucoma	1 (0.3)
Brain imaging	26 (9.8)
Normal	22 (8.3)
Calcification	4 (1.5)
Renal imaging	49 (18.6)
Normal	47 (17.8)
Renal stone	2 (0.8)
24-h urinary Ca	29 (11)
BMD	22 (8.3)
Osteoporosis	1 (0.4)
Osteopenia	7 (2.6)
LBM	1 (0.4)
Normal	13 (4.9)
Tests ordered by endocrinology	36 (12.9)
Others	56 (21.2)

*Note:* Data are presented as number (%). LBM = low bone mass for chronological age.

Abbreviation: BMD = bone mineral density.

**Table 4:** Patients achieved target values.

	Patients achieved target values (*n*)	Target range
Calcium, mg/dL, *n*, (%)	224 (84.8%)	> 8
Phosphorus, mg/dL	196 (74.2%)	< 4.5
Magnesium, mg/dL	261 (98.9%)	1.3–2.7^∗^
CaxP, mg^2^/dL^2^	264 (100%)	< 55
24-h urinary Ca, mg/24 h		< 300 mg/24 h for male < 250 mg/24 h for female
Male	4/6	
Female	13/23	
Complications		
5/5	0	
4/5	0	
3/5	0	
2/5	6	
1/5	12	

^∗^Laboratory reference values in our center.

## Data Availability

The datasets generated and/or analyzed during the current study are not publicly available due to privacy and ethical concerns but are available from the corresponding author on reasonable request.
